# A Ponceau S Staining-Based Dot Blot Assay for Rapid Protein Quantification of Biological Samples

**DOI:** 10.3390/gels8010043

**Published:** 2022-01-07

**Authors:** Dario Lucas Helbing, Leopold Böhm, Nova Oraha, Leonie Karoline Stabenow, Yan Cui

**Affiliations:** 1 Leibniz Institute on Aging—Fritz Lipmann Institute, 07745 Jena, Germany; leopold.boehm@leibniz-fli.de (L.B.); nova.oraha@leibniz-fli.de (N.O.); 2 Faculty of Medicine, Friedrich-Schiller-University Jena, 07743 Jena, Germany; Leonie.Stabenow@med.uni-jena.de; 3 Institute of Molecular Cell Biology, University Hospital Jena, Friedrich-Schiller-University, 07745 Jena, Germany; 4 International Center for Aging and Cancer, Hainan Medical University, Haikou 571199, China

**Keywords:** protein quantification, gel electrophoresis, Western blot, dot blot

## Abstract

Despite the availability of a wide range of commercial kits, protein quantification is often unreliable, especially for tissue-derived samples, leading to uneven loading in subsequent experiments. Here we show that the widely used Bicinchoninic Acid (BCA) assay tends to underestimate protein concentrations of tissue samples. We present a Ponceau S staining-based dot-blot assay as an alternative for protein quantification. This method is simple, rapid, more reliable than the BCA assay, compatible with biological samples lysed in RIPA or 2x SDS gel-loading buffer, and also inexpensive.

## 1. Introduction

Many methods exist for quantifying total protein contents in cell or tissue lysates. However, the conventional colorimetric methods, such as the Bicinchoninic Acid (BCA) [1], Lowry [2] and Bradford [3] assays, have several drawbacks: (1) their requirement for large volumes of samples ranging from 10 to 25 µL per replicate could be difficult to meet for samples with low protein contents, such as mouse nerve or lymph node lysates; (2) colorimetric assays tend to saturate if the protein concentration is too high; in this case, the samples must be diluted for repetition, consuming more materials and time; (3) the widely used SDS-based lysis buffers often contain bromophenol blue, a substance that interferes with most colorimetric assays, making colorimetric assays unsuitable for bromophenol blue-containing lysates [1,4]; and (4) some native components in biological samples, such as high concentrations of lipids in nerve or brain lysates, may interfere with the standard BCA assay [5]. Therefore, protein concentrations of biological samples determined by the common assays are often imprecise, resulting in unequal sample loading in subsequent experiments such as Western blots.

Here, we demonstrate a modified Ponceau S-based dot blot (PDB) assay that has distinct advantages over similar methods described elsewhere [6,7,8] concerning its ability to quantify proteins in cell or tissue lysates, its highly linear standard curve with a wide range from 0.25 to 12 µg/µL, and its overall improved workflow. We further show that protein loading based on the PDB assay was equal in Western blots, essentially eliminating the need for error-prone normalization of immunoblot band intensities to those of a loading control [9]. Therefore, the PDB assay is a reliable method for protein quantification, thereby facilitating subsequent experiments. Our work, in tandem with several other publications, demonstrates the versatility of dot blot in protein quantification and detection [10,11,12].

## 2. Materials and Methods

### 2.1. Reagents

Coomassie Blue solution: 0.05% (*w*/*v*) CBB-R250 (Carl Roth GmbH + Co. KG, Karlsruhe, Germany, #6862), 40% (*v*/*v*) ethanol, 10% (*v*/*v*) acetic acid in water.

Direct Blue solution: 0.008% (*w*/*v*) Direct Blue 71 (Merck KGaA, Darmstadt, Germany, #212407), 40% (*v*/*v*) ethanol and 10% (*v*/*v*) acetic acid in water.

Micro BCA Protein assay kit, Thermo Fisher Scientific Inc., Waltham, MA, USA, #23225.

Pierce Bovine Serum Albumin Standard (BSA) ampules, Thermo Fisher Scientific Inc., Waltham, MA, USA, #23209.

Ponceau S solution: 0.1% (*w*/*v*) Ponceau S (Merck KGaA, Darmstadt, Germany, #P3504-10G) in 5% (*v*/*v*) acetic acid.

RIPA buffer: Pierce RIPA buffer (Thermo Fisher Scientific Inc., Waltham, MA, USA) or self-made RIPA buffer (2.5% (*v*/*v*) 1 M Tris-HCl, 0.88% (*w*/*v*) NaCl, 1% (*w*/*v*) NP-40, 1% (*w*/*v*) sodium deoxycholate, 0.1% (*w*/*v*) SDS in water, adjusted to pH 7.6), 1 tablet/50 mL cOmplete protease inhibitor (Roche Diagnostics GmbH, Mannheim, Germany), 1 tablet/10 mL RIPA phosSTOP phosphatase inhibitor (Roche Diagnostics GmbH, Mannheim, Germany).

2x SDS Gel Loading buffer (2x SDS LB): 0.1 M Tris-HCl, 4% (*v*/*v*) SDS, 20% (*v*/*v*) Glycerol, 0.2% (*v*/*v*) Bromophenol blue (Carl Roth GmbH + Co. KG, Karlsruhe, Germany, #A512.1) in water, adjusted to pH 6.8.

### 2.2. Ethics Statement

The Thuringian State Office for Consumer Protection (TLV), Department of Health and Technical Consumer Protection has examined and prospectively approved the animal experiment application under the permit number 03-046/16. It is advised by the Thuringian Animal Protection Commission.

### 2.3. Experimental Animals

Mice (C57BL/6J) were housed under a 12 h dark/light cycle and had free access to food and water. All mice were handled in strict adherence to local governmental and institutional animal care regulations and euthanized by trained personnel using CO_2_ inhalation. All efforts were made to minimize suffering.

### 2.4. Lysate Preparation

Cell lysates were prepared by adding 2x SDS LB to cell culture plates after washing off cell debris and culture media and then collected with cell scrapers. Lysates were then transferred to Eppendorf tubes and vortexed for 10 s.

Sciatic nerves, brains and spleens were harvested from euthanized mice and lysed using ceramic beads in a Precellys 24 homogenizer (Bertin Instruments, Montigny-le-Bretonneux, France) in either RIPA buffer or 2x SDS LB.

### 2.5. BCA Assay

The microscale BCA assay was performed according to the manufacturer’s instruction. Protein concentrations were calculated using the linear equation based on the trend line of the standard curve generated with Microsoft Excel.

### 2.6. Dot Blot, Ponceau S Staining, Direct Blue Staining and Coomassie Blue Staining

Lysates in 2x SDS LB were boiled for 8 min at 98 °C beforehand. Different volumes of lysates and BSA solution were applied dot-wise to dry nitrocellulose membranes. Membranes were then left to dry for 15 min. Membranes with samples lysed in 2x SDS LB were then washed three times (5 min each) in deionized (DI) water on a shaker before staining with Ponceau S solution for 1 min, and membranes with samples lysed in RIPA buffer were stained directly with Ponceau S solution for 1 min. Membranes were then briefly washed with DI water to remove the Ponceau S solution. Membranes were then placed into a plastic film and scanned with an Epson Perfection V750 Pro scanner.

For re-staining of membranes with Direct Blue solution, membranes previously stained with Ponceau S were washed overnight with TBS-T, stained with Direct Blue staining solution for 5 min, washed with a solution of 40% (*v*/*v*) ethanol and 10% (*v*/*v*) acetic acid in DI water to remove non-specific background staining, and then imaged as described above. For re-staining of membranes with Coomassie Blue, membranes previously stained with Direct Blue were washed overnight with a solution of 50% (*v*/*v*) ethanol and 15% (*w*/*v*) 1 M sodium bicarbonate in water. Membranes were then stained with Coomassie Blue solution for 3 min and imaged as described above.

For experiments using diluted BSA solutions, a BSA solution was diluted 1:1 with either 0.5 M NaCl, PBS, TBS-T or a 0.1% (*v*/*v*) SDS solution, resulting in BSA solutions with a protein concentration of 1 µg/µL. BSA solutions were applied dot-wise onto a nitrocellulose membrane with different volumes to create a standard curve for each solution based on Ponceau S staining.

We included a movie demonstrating the experimental workflow and the analysis with Fiji in the Supplementary Materials (Video S1).

### 2.7. Protein Quantification with Fiji and Microsoft Excel

After transforming a source image into an 8-bit greyscale image and inverting it in ImageJ/Fiji, the rectangle tool and ROI manager were used to define a series of regions of interest (ROIs), covering each dot with a single ROI of the same size, and the integrated density of each ROI was measured. The integrated density values of technical replicates of the samples and the standards were averaged and, if necessary, corrected for the baseline signal by subtracting the trendlines’ y-axis intercept. A standard curve was generated based on the mean integrated density values of the standards and the equation of a corresponding linear trendline used to calculate protein concentrations. The goodness of fit/coefficient of determination for each trendline was calculated in Microsoft Excel and displayed as R^2^ in the corresponding figure.

### 2.8. Cost Calculations

We calculated the costs regarding the PDB assay and the micro BCA assay for measuring 12 biological samples and standards. Both the commercial BCA assay and a self-made variant were compared. The reagents for a self-made BCA assay were as follows:

Reagent A: 1% (*w*/*v*) sodium bicinchoninate, 2% (*w*/*v*) sodium carbonate, 0.16% sodium tartrate (*w*/*v*), 0.4% (*w*/*v*) NaOH, 0.95% (*w*/*v*) sodium bicarbonate in water, pH adjusted to 11.25.

Reagent B: 4% (*w*/*v*) cupric sulfate in water.

A range of total costs was calculated for each method to reflect the market price ranges of the required materials.

We calculated the costs of RIPA buffer and SDS Gel loading buffer per ml and compared the commercial and self-made RIPA buffers with the self-made SDS Gel loading buffer.

We determined the prices of reagents on the websites of established distributors and used the smallest available package size for our calculations. Basic laboratory chemicals such as Tris-HCl or NaCl were not included in our calculations. We included the protease and the phosphatase inhibitor prices in both cost calculations for the commercial and the self-made RIPA buffers.

### 2.9. Immunoblotting

Immunoblotting was performed as previously described [13]. Antibodies are listed in Table 1. Blots were developed with Pierce ECL Western Blotting Substrate (Thermo Fisher Scientific Inc., Waltham, MA, USA).

### 2.10. Statistical Procedures and Figure Preparation

The *p*-values were calculated by Student’s *t*-test with Graphpad Prism 7.0. Statistical significance was accepted at *p* ≤ 0.05. All data are presented as mean +/− SEM (Standard error of the mean). Missing error bars are due to technical reasons when the error bar would be shorter than the height of a single datapoint. All figures were made with Graphpad Prism 7.0 and assembled in Adobe Photoshop CS6 or Microsoft PowerPoint.

## 3. Results and Discussion

**Figure 1:**
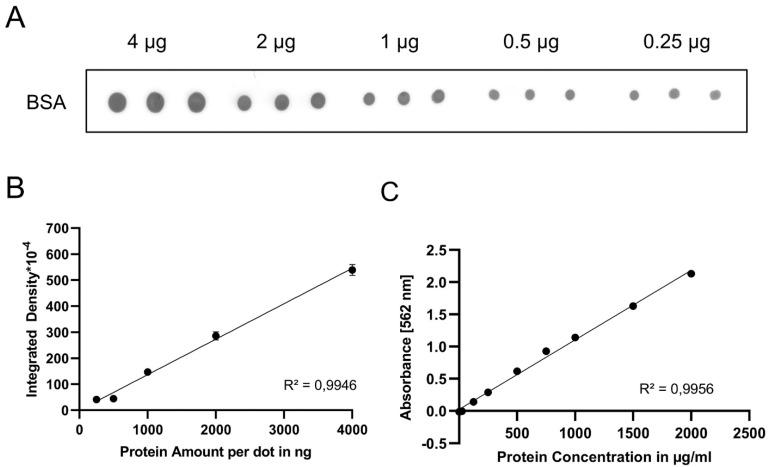
Standard curves of the PDB and the BCA assays. (**A**) Ponceau S-stained dot blot. Variable volumes of a BSA solution (2 µg/µL) containing indicated BSA amounts were spotted in triplicate onto a nitrocellulose membrane. (**B**) Inverted standard curve based on Figure 1A. The experiment was repeated with three batches of BSA solutions. (**C**) Standard curve of three BCA assays performed with different BSA solutions. Error bars indicate SEM. R^2^ = Coefficient of determination.

To assess the reliability of the PDB assay for protein quantification, we spotted different volumes of a BSA solution (0.25–4 µg of BSA) on a nitrocellulose membrane. Ponceau S staining was performed after air-drying the membrane (Figure 1A). The sizes of the dots did not correlate with the total protein amounts (data not shown) and we therefore measured the integrated density, which is the sum of grey values in the region of interest. It should be noted that a gray value measured by ImageJ is opposite to the actual intensity, so images should be inverted before measurement. The integrated density and protein amount showed a linear trendline, indicated by a coefficient of determination *R^2^* near the optimal value of 1 (Figure 1B). For comparison, we performed a BCA assay, which also showed a trendline with R^2^~1 (Figure 1C), indicating that both assays perform well within their respective detection range. However, according to the manufacturer, beyond the maximum concentration of 40 µg/mL, the BCA assay—like any other colorimetric assay—is no longer reliable and further dilution of the sample is required. In contrast, the PDB assay yielded a linear standard curve up to 12 µg of the protein per dot (Figure S1).

**Figure 2:**
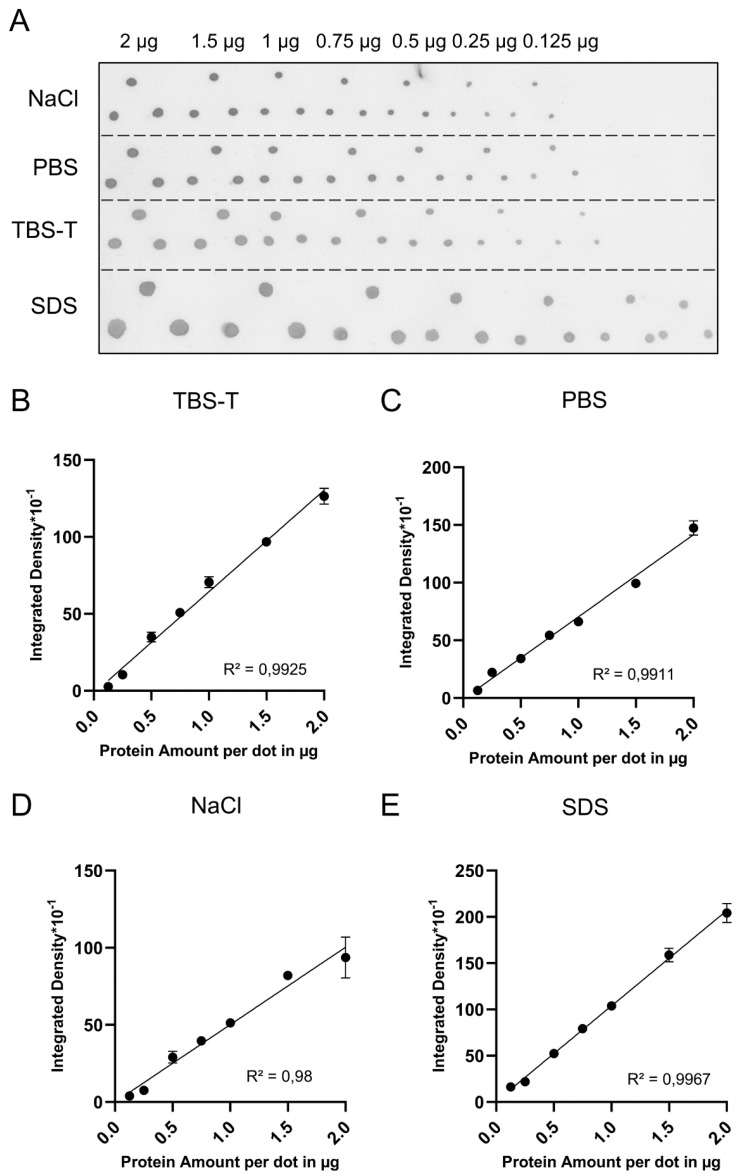
Comparison of diluents for the PDB assay. (**A**) Ponceau S-stained dot blot. BSA was diluted in different solutions to a final concentration of 1 µg/µL. Variable volumes of the solutions containing indicated BSA amounts were spotted in triplicate onto a nitrocellulose membrane. (**B**–**E**) Inverted standard curves based on Figure 2A. Error bars indicate SEM. R^2^ = Coefficient of determination.

Visible in Figure S1, the bottom range of the PDB assay standard curve may lose fidelity if the total protein amount is below 1 µg and the respective BSA volumes are not spotted perfectly, which can occur if the applied volume per drop is small. Therefore, the BSA standards may be diluted if the samples are suspected of having low protein contents. To identify suitable diluents, we diluted the BSA standards with TBS-T, 0.1% SDS, PBS and 0.5 M NaCl, and compared the corresponding PDB assay standard curves (Figure 2).

As indicated by higher R^2^ values and lower error deviations between technical replicates, TBS-T and 0.1% SDS in water outperformed PBS and 0.5 M NaCl (Figure 2B–E). Using diluted standards, samples with very low protein concentrations just above 0.125 µg/µL can be analyzed.

As described above, fixing the protein concentration while varying the volume yielded a linear standard curve in the PDB assay. Similarly, fixing the volume while varying the protein concentration also yielded a linear standard curve (Figure S2), suggesting that the integrated density (if not saturated) is determined by the protein amount and less affected by the dot size.

**Figure 3:**
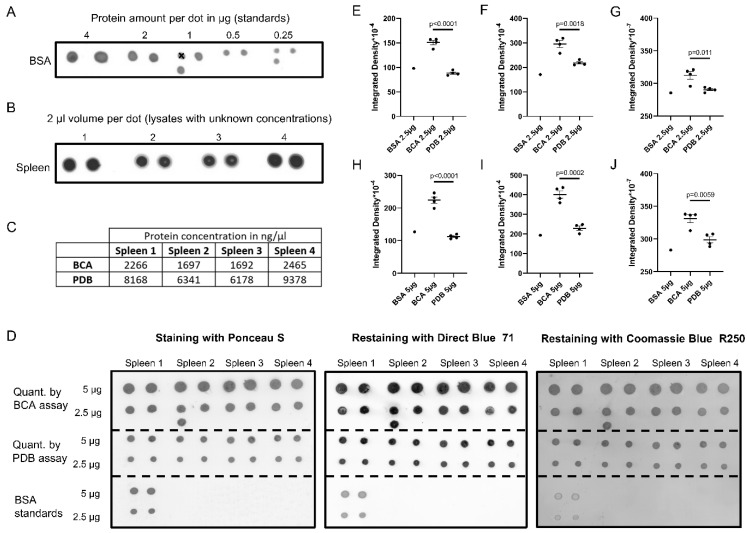
Underestimation of protein concentrations by the BCA assay. (**A**) Ponceau S-stained dot blot. Variable volumes of a BSA solution (2 µg/µL) containing indicated BSA amounts were spotted onto a nitrocellulose membrane. A cross indicates an incorrectly applied sample that was excluded for the analysis. (**B**) Ponceau S-stained dot blot. A quantity of 2 µL of spleen lysates from four mice was spotted in duplicates onto a nitrocellulose membrane. (**C**) Calculated protein concentrations of the spleen samples based on either the PDB or the BCA assay. (**D**) Retest of the protein concentrations of the spleen samples by different staining of the dot blot. Spleen samples containing 5 or 2.5 µg of total proteins (calculation based on either the PDB or the BCA assay), in addition to the BSA standards (2 µg/µL), were spotted in duplicate onto a nitrocellulose membrane. The blot was sequentially stained with Ponceau S, Direct Blue 71 and Coomassie Blue R250, with destaining between stainings. The integrated density values were then plotted: (**E**,**H**) for Ponceau S staining; (**F**,**I**) for Direct Blue 71 staining; (**G**,**J**) for Coomassie Blue R250 staining. Error bars indicate SEM. *p*-values were calculated using Student’s *t*-test.

To test whether the PDB assay can be used to quantify proteins in lysates of biological tissues, spleens from four mice were lysed in 1 mL of RIPA buffer and a PDB assay was performed (Figure 3A–C). For comparison, the same lysates were also measured with a BCA assay (Figure 3C).

Both standard curves had to be extrapolated in order to calculate the protein concentrations of the spleen lysates because of high protein contents, which should generally be avoided. The BCA assay showed sample concentrations above the range of the respective standard curve. It would be necessary to repeat the measurement either with diluted samples or with another serial of BSA standards. In contrast, the calculation of the protein concentrations based on the PDB assay is less problematic because the standard curve for the PDB assay shows a linear correlation until 12 µg total protein amount per dot (Figure S1). It should be mentioned that, in routine experiments where the protein concentrations are estimated to be low, such high standards might not be necessary. 

Strikingly, the protein concentrations of the spleen lysates based on the PDB assay were three to four times higher than those determined by the BCA assay (Figure 3C). To validate the results, we performed a PDB assay spotted with 5 and 2.5 µg of total proteins, which was based on the concentrations determined by either the BCA or the PDB assay (Figure 3D, left panel). Comparison of the integrated densities revealed a higher variability of the dots based on the BCA assay (Figure 3E,H). Importantly, the integrated densities of the dots based on the BCA assay were generally much higher than those of the BSA standards. In contrast, the integrated densities of the dots based on the PDB assay were similar to those of the BSA standard (Figure 3E,H). This retest suggests that the PDB assay is more reliable than the BCA assay, whereas the latter may significantly underestimate the total protein contents of unknown samples

Protein inputs on blots can also be stained with other dyes. Different dyes may have different binding modes to proteins. Unequal binding to heterogenous proteins will lead to a bias in total protein quantification. Ponceau S binds to positively charged amino acids and nonpolar regions of proteins [14]; therefore, it stains heterogenous proteins relatively equally. To compare Ponceau S staining with two other staining methods, we destained the same blot and re-stained it with Direct Blue 71 [15] and Coomassie Blue R250 sequentially. The overall staining profiles and integrated densities were comparable between the three dyes, despite the fact that the BSA standards were less stained by Direct Blue 71 or Coomassie Blue R250 (Figure 3D–J). It is unclear whether this difference was due to each dye’s binding preference or loss of BSA from the blot during extensive destaining. It is also apparent that Coomassie Blue R250 staining resulted in a high background. Due to its properties of having a simple recipe, fast procedure, relatively equal staining of heterogenous proteins, adequate sensitivity, and low background, Ponceau S staining is an excellent choice for protein quantification.

We have shown that the PDB assay is compatible with biological tissues lysed in RIPA buffer, but some researchers may prefer 2x SDS LB because some proteins are known to be insoluble in RIPA buffer, leading to an overall loss of 10% to 30% of all proteins [9]. We therefore tested the compatibility of the PDB assay with samples lysed in 2x SDS LB. Because an initial experiment with BSA diluted in 2x SDS LB did not yield a linear standard curve (data not shown), we decided to add a washing step to wash out the SDS from the dots, which we suspected interfered with the binding of Ponceau S to the proteins. Washing the membrane three times with DI water (5 min each) after drying it for 15 min allowed us to generate a standard curve similar to that based on undiluted BSA standards (Figure S3), allowing the PDB assay to measure samples lysed in 2x SDS LB. RIPA buffer emerged to be unsuitable to dilute BSA standards because it results in the formation of coffee rings on blots (Figure S4).

**Figure 4:**
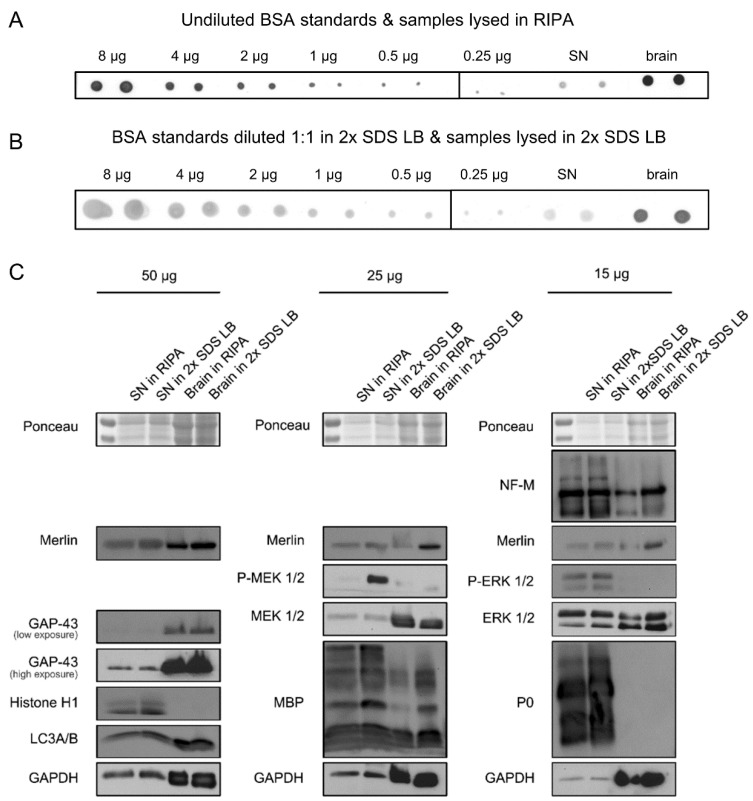
Western blots based on protein quantification with the PDB assay. (**A**,**B**) Ponceau S-stained dot blot. Undiluted BSA standards and BSA standards diluted 1:1 in 2x SDS lysis buffer were spotted in duplicate onto a membrane (fixed concentration, variable volumes). A quantity of 1 µL of sciatic nerve (SN) and brain samples lysed in 2x SDS LB or RIPA buffer was also applied onto the same membrane for quantification with the PDB assay. (**C**) The nerve and brain lysates containing 50, 25 or 15 µg of total proteins (based on the PDB assay) were loaded for SDS-PAGE and Western blot. After protein transfer to a nitrocellulose membrane, the membrane was also stained with Ponceau S.

To compare the protein extraction efficiency between 2x SDS LB and RIPA buffer, we performed a PDB assay with tissues lysed in these two buffers and conducted a subsequent Western blot. Two sciatic nerves and cerebral hemispheres, each from one mouse (two mice in total), were pooled and lysed. Due to their high lipid content, both tissues were hard to lyse. We measured the total protein contents of the lysates by the PDB assay, and then loaded the lysates containing 50, 25, and 15 µg of total proteins for SDS-PAGE and Western blot (Figure 4C).

Ponceau S staining of the membrane after protein transfer revealed equal loading of the samples (Figure S5), indicating that the PDB assay performs similarly as lysates in RIPA buffer or 2x SDS LB. Interestingly, we observed striking differences in the band intensities between the nerve and the brain lysates. We hypothesize that this is due to a high abundance of albumin (the strong band below 70 kDa) and the IgG heavy (the strong band slightly above 55 kDa) and light chain (the strong band between 25 and 35 kDa) in the peripheral nerves, which are absent in the brains because of the blood–brain barrier [16,17]. This circumstance will lead to an overestimation of the actual protein content of the sciatic nerve and thus biased loading. This could be a major issue when comparing protein expression between the peripheral and the central nervous systems.

Our Western blot results showed that the cytoskeleton-associated protein merlin, as well as the nuclear protein Histone H1, the autophagic vesicle membrane proteins LC3A/B, and the myelin basic protein MBP, were better extracted by 2x SDS LB than by RIPA buffer (Figure 4C). In contrast, the cytoplasmic proteins MEK 1/2, ERK 1/2, GAPDH, and GAP-43 were indistinguishably extracted by the two buffers. These findings confirm the literature reports that 2x SDS lyses biological tissues more effectively than RIPA buffer, which can be attributed to the insolubility of cytoskeleton-associated and extracellular matrix proteins in RIPA [12]. 

To our surprise, p-ERK1/2 and p-MEK1/2 levels were higher in 2x SDS LB lysates than in RIPA lysates. We could not exclude the possibility that p-ERK1/2 and p-MEK1/2 in the respective nerves were indeed different. If this is not the case, the result suggests that RIPA buffer, which contains a designated phosphatase buffer, is inferior to 2x SDS LB in terms of phosphatase inhibition, which may be explained by the strong denaturing effect of SDS. This observation may be important for studying rapidly changing signaling processes, such as degeneration and regeneration in the nervous system [18,19], where 2x SDS LB may be a better choice for tissue lysis.

In the course of our experiments, we found that the PDB assay, especially when using 2x SDS LB instead of RIPA buffer to lyse biological tissue, can be much cheaper than the established workflow in our laboratory, which relies on the widely used BCA assay for protein quantification. Therefore, we calculated the costs for quantification of 12 samples with a commercial or a self-made BCA assay, or a PDB assay. In this study, we only used the commercial BCA assay, and the reagents can be self-made. The self-made BCA kit would cost between EUR 15.29 and 24.91, a commercial BCA kit was EUR 13.47, and the PDB assay cost only EUR 2.05 for quantification of 12 samples. From our experience, Ponceau S solution can be reused at least 20 times to stain transfer membranes or dot blots. A laboratory quantifying 1000 samples with the PDB instead of the BCA assay would save about EUR 950 (Figure S6). Because we have shown that direct lysis of biological tissues in 2x SDS LB is compatible with protein quantification by PDB assay and superior to RIPA buffer in terms of protein solubilization, we also checked the overall price difference between RIPA and 2x SDS LB, which amounted to a difference of between EUR 1380 and 2310 for 1000 lysates (Figure S7). One laboratory could save over EUR 2300 per 1000 lysates by switching from tissue lysis with RIPA buffer and protein quantification with the BCA assay to 2x SDS LB and our PDB assay.

The time needed for a PDB assay or a BCA assay is comparable: both assays will take around 45 min from sample preparation to measurement with a plate reader (BCA assay) or to scanning (PDB assay); the subsequent analysis including standard curve preparation, linear regression, and calculation of protein concentrations is almost the same.

Previously, Morcol et al. described a similar method to quantify purified proteins [8]. Here, we demonstrate an improved PDB assay that can be used to quantify tissue lysates and is compatible with 2x SDS LB. The PDB assay has the following advantages: (1) it can be easily adjusted to quantify samples with very high or low protein contents; (2) it requires far fewer materials than the BCA assay, saving valuable samples; (3) it only needs a simple laboratory equipment; and (4) it is significantly cheaper and more reliable than the BCA assay, thereby facilitating downstream experiments relying on correct sample inputs. 

## 4. Conclusions

The PDB assay is a cheap and reliable method for quantifying proteins in tissue lysates.

## Figures and Tables

**Table 1:** List of antibodies.

Antibody	Host Species	Supplier	Cat. No.	Dilution
ERK 1/2	Mouse	Cell Signaling	4696	1:2000
GAP-43	Rabbit	Santa Cruz	10,786	1:500
GAPDH	Mouse	Santa Cruz	32,233	1:5000
Histone H1	Mouse	Santa Cruz	8030	1:500
LC3A/B	Rabbit	Cell Signaling	4108	1:1000
MBP	Rat	Novus Biologicals	NB600-717	1:1000
MEK 1/2	Rabbit	Cell Signaling	8727	1:2000
Merlin	Rabbit	Cell Signaling	12,896	1:1000
NF-M	Mouse	Santa Cruz	16,143	1:500
P0	Chicken	Abcam	39,375	1:2000
p-ERK 1/2	Rabbit	Cell Signaling	4370	1:2000
p-MEK1/2	Mouse	Abcam	91,545	1:2000
Anti-chicken HRP	Goat	Abcam	97,135	1:5000
Anti-Rabbit HRP	Goat	Agilent Dako	P0448	1:2000
Anti-Mouse HRP	Goat	Agilent Dako	P0447	1:2000
Anti-rat HRP	Rabbit	Invitrogen	61-9520	1:2000
Anti-Goat HRP	Rabbit	Agilent Dako	P0449	1:1000

## Data Availability

All data underlying the results are available as part of the article and no additional source data are required.

## Supplementary Materials

The following are available online at https://www.mdpi.com/article/10.3390/gels8010043/s1. Figure S1: Extended range for the PDB assay; Figure S2: The integrated density is determined by the protein amount instead of the dot size; Figure S3: Standard curves related to Figure 4A,B; Figure S4: BSA standards diluted with RIPA buffer forms “coffee rings” on dot blots; Figure S5: Ponceau S staining of the membrane after SDS-PAGE and protein transfer, uncropped, related to Figure 4C; Figure S6: Cost comparison between the PDB and the BCA assays; Figure S7: Cost comparison between the PDB assay with samples lysed in 2x SDS LB and the BCA assay with samples lysed in RIPA buffer; Video S1: Workflow of the PDB assay.
