# Supplementary material for: A Ponceau S Staining-Based Dot Blot Assay for Rapid Protein Quantification of Biological Samples
Source: Gels. 2022 Jan 7;8(1):43. doi: 10.3390/gels8010043 (PMC8775029; doi:10.3390/gels8010043)
Supplement: Supplementary file 1 [file gels-08-00043-s001.zip › gels-1506830-supplementary.pdf]

Supplementary materials

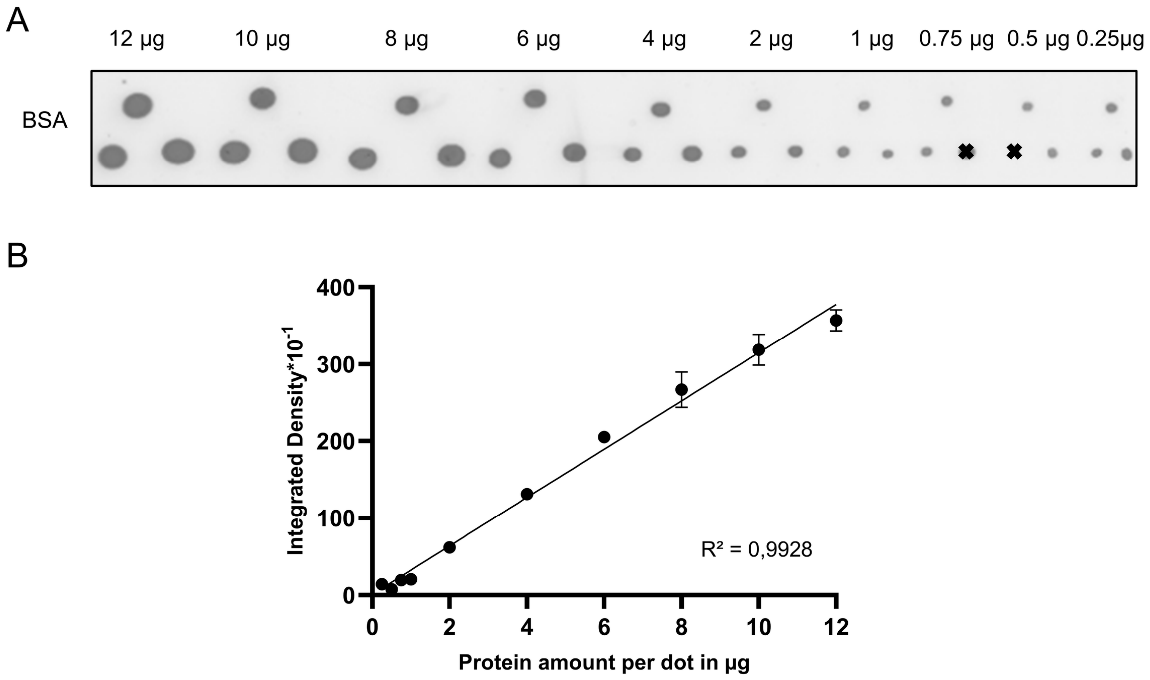

Figure S1. Extended range for the PDB assay. **A.** Ponceau S-stained dot blot. BSA standards (fixed concentration) were spotted in triplicates onto a nitrocellulose membrane. **B.** Standard curve based on the integrated density values of the BSA standards in A.

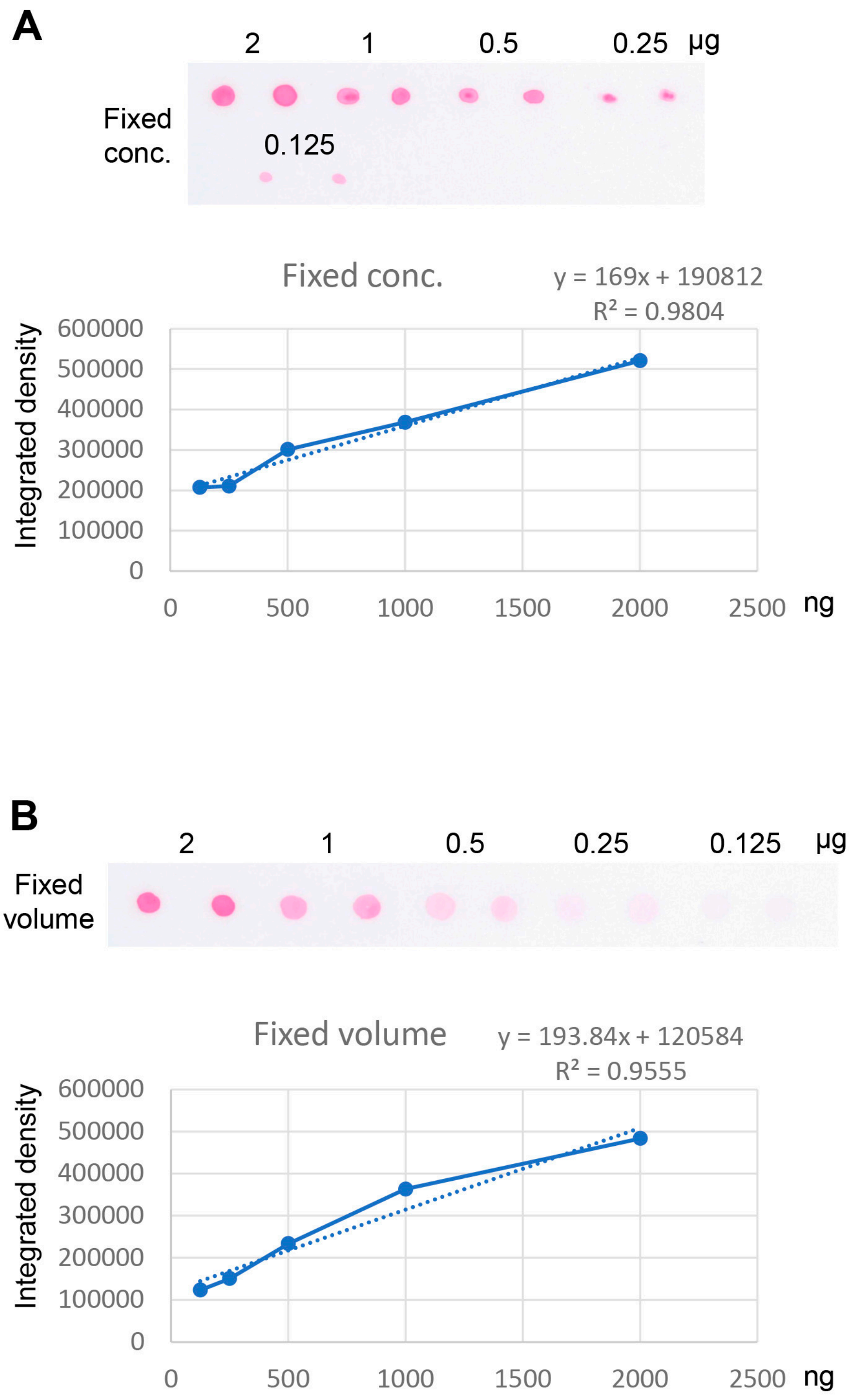

Figure S2. The integrated density is determined by the protein amount instead of the dot size. A. Standard curve based on the BSA standards with fixed concentration. **B.** Standard curve based on the BSA standards with fixed volume. The BSA standards were diluted with 0.1% SDS/H_2O, and spotted in duplicates.

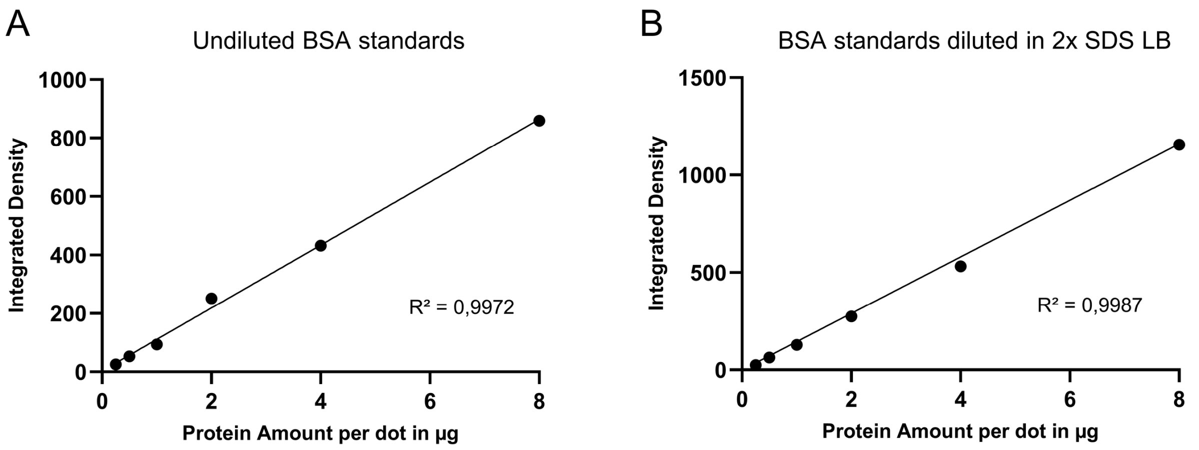

Figure S3. Standard curves related to Figure 4 A, B**. A.** Standard curve of a PDB assay performed with undiluted BSA standards (related to Fig 4A). **B.** Standard curve of a PDB assay performed with BSA standards diluted 1:1 in 2x SDS LB (related to Fig 4B). The BSA standards were ranging from 8 µg to 0.5 µg of total protein amount per dot.

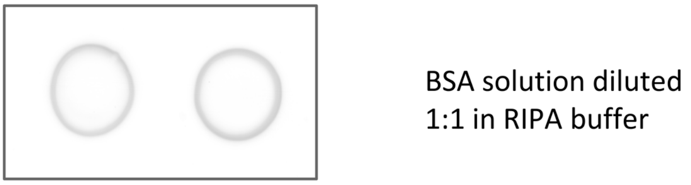

Figure S4. BSA standards diluted with RIPA buffer forms "coffee rings" on dot blots.

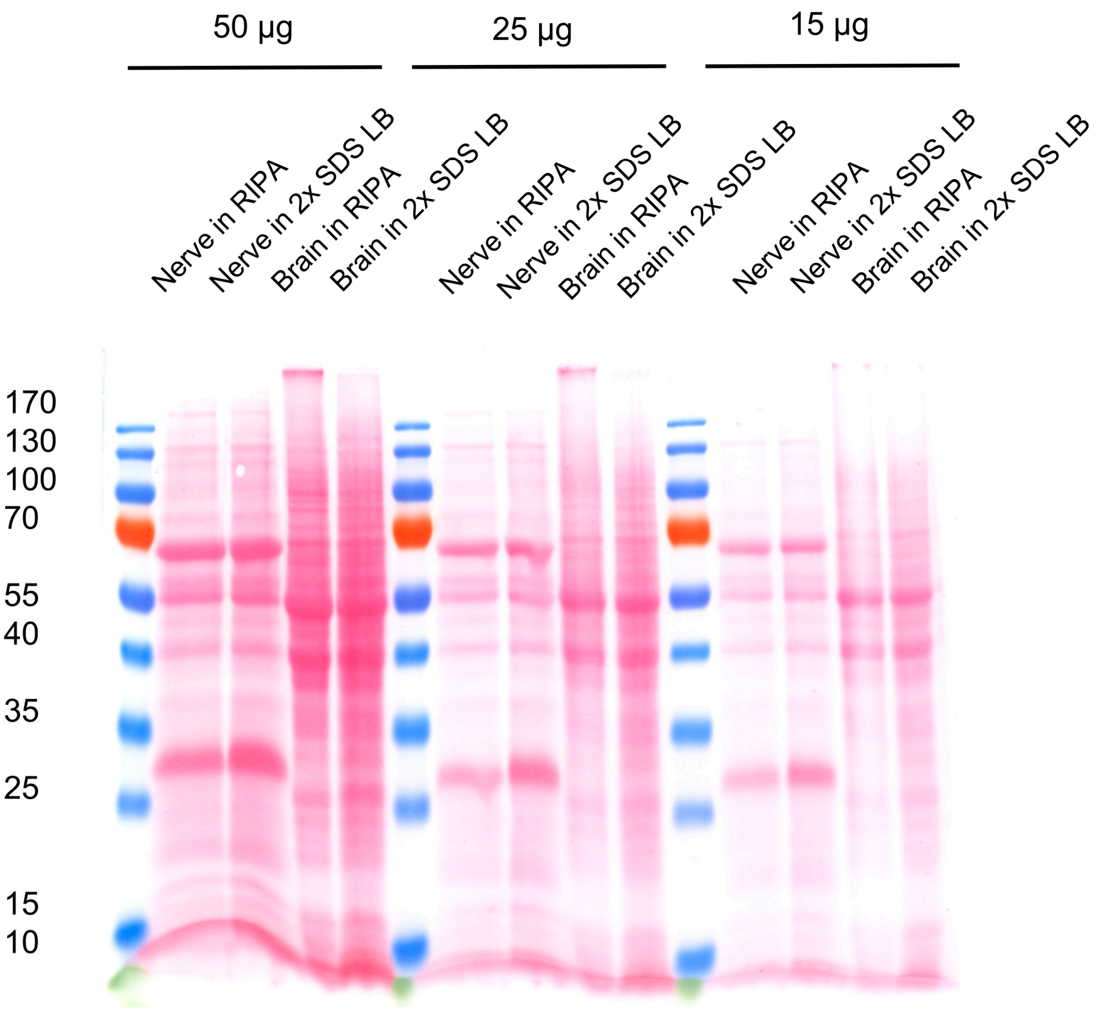

Figure S5. Ponceau S staining of the membrane after SDS-PAGE and protein transfer, uncropped, related to Fig. 4C. The loading of the nerve and the brain lysates was based on protein quantification with the PDB assay.

**Method**	**Materials**	**Price (for 12 samples)**
Ponceau Dot Blot	• Ponceau powder<br>• Nitrocellulose membrane (84cm^2)<br>• Bovine serum albumin	2,05€
Commercial BCA Kit	• Kit Materials<br>• Pierce 96-wellplate	13,47€
Selfmade BCA Kit	• Kit Materials<br>• Pierce 96-wellplate	Ranging from 15,29€ to 24,91€ (depending on producer)
	Price for 1000 samples<br>Ponceau Dot Blot: 170,83€<br>Commercial BCA Kit: ~1122,50€<br>**Δ= ~951,67€**	

Figure S6. Cost comparison between the PDB and the BCA assays.

Lysis buffer	Price per sample in 1 ml lysis buffer	Cat.Nr./Supplier
cOmplete Protease Inhibitor Cocktail	0,28€	000000011697498001/Roche
PhosStop	1,20€	000000004906845001/Roche
RIPA buffer (commercial)	0,93€	89900/Thermo Scientific
RIPA buffer, final solution	2,41€	
cOmplete Protease Inhibitor Cocktail	0,28€	000000011697498001/Roche
PhostStop	1,20€	000000004906845001/Roche
RIPA buffer (selfmade)	0,0006€	Various suppliers
RIPA buffer, final solution	1,48€	
SDS gel loading buffer (selfmade)	0,1€	Various suppliers
	**Price for 1000 lysates**<br>RIPA buffer: 2410€<br>RIPA buffer (selfmade): 1480€<br>2x SDS LB: 100€<br>**Δ=1380 to 2310€**	
	**Price for 1000 lysates+quantification**<br>Commercial BCA kit with lysates in either selfmade or commercial RIPA buffer: ~2602,5 to 3532,5€<br>Ponceau Dot Blot Assay with lysates in 2x SDS LB: 270,83 €<br>**Δ= 2331,67 to 3261,67€**	

Figure S7. Cost comparison between the PDB assay with samples lysed in 2x SDS LB and the BCA assay with samples lysed in RIPA buffer.

Video S1. Workflow of the PDB assay.
